# Effects of *Lacticaseibacillus rhamnosus* LOCK900 on Development of Volatile Compounds and Sensory Quality of Dry Fermented Sausages

**DOI:** 10.3390/molecules26216454

**Published:** 2021-10-26

**Authors:** Barbara Sionek, Krzysztof Tambor, Anna Okoń, Piotr Szymański, Dorota Zielińska, Katarzyna Neffe-Skocińska, Danuta Kołożyn-Krajewska

**Affiliations:** 1 Institute of Human Nutrition Sciences, Warsaw University of Life Sciences (WULS), Nowoursynowska St. 159C, 02-776 Warszawa, Poland; barbara_sionek@sggw.edu.pl (B.S.); krzysztof_tambor@sggw.edu.pl (K.T.); dorota_zielinska@sggw.edu.pl (D.Z.); danuta_kolozyn_krajewska@sggw.edu.pl (D.K.-K.); 2 Prof. Wacław Dąbrowski Institute of Agricultural and Food Biotechnology—State Research Institute, Rakowiecka St. 36, 02-532 Warsaw, Poland; anna.okon@ibprs.pl (A.O.); piotr.szymanski@ibprs.pl (P.S.)

**Keywords:** volatile compound analysis, GC–MS method, sensory profile, flavour and odour, probiotic, *Lacticaseibacillus*, functional food

## Abstract

Traditional dry fermented meat products are highly appreciated by consumers. A probiotic starter culture increases their attractiveness through sensory qualities and a potential health-promoting effect. The ability to scale the laboratory solution to industrial conditions is an additional scientific and practical value of a new way of using probiotics in the meat industry. The aim was to evaluate the influence of the probiotic starter culture *Lacticaseibacillus rhamnosus* LOCK900 on the development of volatile organic compounds and the sensory quality of dry fermented pork sausages during fermentation and refrigeration storage. The microbiological and sensory characteristic (QDA method) and volatile compound (gas chromatography coupled with mass spectrometry: GC–MS) were evaluated. The number of LOCK900 cells during 12 weeks of storage remained above 6 log CFU g^−1^, making this product a functional food. The addition of probiotic LOCK900 increased the levels of acidic volatile compounds, aldehydes, and esters, which, combined with the additives and spices used, had a positive effect on the sensory properties of ripening sausages. The sausages with LOCK900 were characterised by positive sensory features, and their overall quality remained high during storage and did not differ from that of the control sausages.

## 1. Introduction

Fermented meat products are appreciated by consumers, among other things, for their characteristic flavour and odour, as well as traditional production methods. An additional value may be the addition of probiotic and potentially probiotic strains of bacteria. The potential pro-health effects can be attractive for consumers. However, there is a lack of studies assessing the effect of probiotic strains on the desired characteristics of dry fermented meat products, such as the sensory quality, storage time, and potential health-promoting effects. The most frequently used quality assessment is sensory analysis and the analysis of volatile organic compounds (VOCs) in the product. Volatile compounds are crucial for the odour and flavour of dry fermented meat [1]. They can arise at various stages of production, and their presence is the result of complex metabolic processes and the use of flavour additives [2,3]. The metabolic processes that take place in dry fermented meat upon the addition of bacteria, such as *Lactobacillus* spp., change the ingredients and composition of compounds in the maturing meat. The enzymes of probiotic bacterial strains transform fats, proteins, and carbohydrates, and degrade amino acids, thus affecting the flavour and odour characteristics of the product [2,4]. The taste and aroma characteristics of a fermented meat product are not only the result of flavour additives, technological processes, and the presence and content of individual substances but also depend on the interaction between these factors and the threshold of perception by consumers [1,3,5].

The present study is a continuation of the research of the optimised technology of the production process of the probiotic starter culture, and the method of adding it to meat products guarantees the survival of the probiotics in the non-sterile meat environment. The natural lactic acid bacteria residing in it may interfere with the results of microbiological analyses in order to determine the amount of added probiotic starter culture. The ability to scale the laboratory solution to industrial conditions is an additional scientific and practical value of a new way of using probiotics in the meat industry [6].

The aim of this study was to evaluate the influence of the probiotic starter culture *Lacticaseibacillus rhamnosus* LOCK900 on the development of volatile compounds and the sensory quality of dry fermented pork sausages during fermentation and refrigeration storage.

## 2. Results and Discussion

### 2.1. Microbiological and pH Evaluation

In the present study, the number of LAB cells in probiotic sausages after fermentation and during storage was approximately 8.0 log CFU g^−1^ (Table 1). In the control sausages, the number of LAB cells was approximately 6.0 log CFU g^−1^, which increased by 1 log level, reaching a value of 7.0 log CFU g^−1^ in the 12th week. According to the FAO/WHO definition, for a probiotic foodstuff, the number of probiotic bacterial cells should be higher than 10^6^ per gram of the product [7]. It is known that raw meat contains some amount of lactic acid bacteria, which can provide a spontaneous fermentation process. However, to obtain a probiotic product, probiotic bacteria should dominate and a high count of these beneficial microorganisms should be achieved. In the previous study, we proved that *Lacticaseibacillus rhamnosus* LOCK900 is able to grow and dominate in meat products [6]. The shredded, non-sterile meat in sausages seems to be a suitable environment for the development of probiotics [8,9,10].

However, technological processes, such as smoking or the addition of spices, may have an adverse effect on the survival of these microorganisms [11]. The pH values of the probiotic sausages were 4.95, 4.88, and 4.70 after production and 6 and 12 weeks of storage, respectively (Table 2), and were lower than those in the control sausages. A decrease in the pH value combined with an increase in the LAB content may inhibit the development of unfavorable microflora because some bacteria do not tolerate the presence of organic acids and pH values below 5.0 [12]. Wójciak et al. [13] also found a clear effect of the use of *Lactobacillus casei* LOCK900 on fermented sausage as the probiotic samples were characterised by higher pH values compared with those of the control samples. The selection of an appropriate starter LAB strain for the technology plays a significant role in the production process of fermented sausages. A relatively low pH, which inhibits the development of unfavorable microorganisms, including pathogenic microorganisms, limits the formation of biogenic amines and improves product safety [14,15,16].

### 2.2. The Composition of Volatile Compounds

A total of 62 volatile organic compounds were identified in the sausages and grouped according to the chemical classes, as shown in Table 3, including two acids, four alcohols, six aldehydes, one amine, and one aromatic hydrocarbon, eight esters, one glycol, five ketones, two phenols, six sulfur compounds, and twenty-six terpenes. Most of the identified compounds have been previously reported in dry fermented sausages [17,18,19].

In meat, volatile organic compounds are produced by the metabolic processes of proteins and fats, mainly oxidation and esterification. During the fermentation and storage of meat, acetic, propionic, and butyric acids, acetaldehyde, diacetyl, and acetoin are formed as the result of lactic acid metabolism. Aldehydes, acids, and alcohols are the products of amino acids metabolism. The free fatty acids in meat products are degraded during the fermentation processes by microbial enzymes and are converted into carbonyl compounds, such as aldehydes, methyl ketones, hydrocarbons, and alcohols [20]. The presence of LAB enzymes leads to the hydrolysis of proteins, polypeptides, and free amino acids, which are aromatic compounds or their precursors [4]. The metabolic reactions in dry fermented meat and flavour additives, such as pepper, garlic, and other spices, are critical to the typical sensory characteristics of both odour and flavour [21,22].

In our study, the probiotic sausages showed a significantly higher level of acidic volatile compounds, mainly acetic acid, compared with that in the control samples both after fermentation and during storage, which, in the sensory assessment, contributed to a more intense sour flavour. The fermentation processes of carbohydrates taking place in meat products due to the presence of LAB increase the content of lactic and acetic acids, causing a decrease in the pH [22]. The increase in the acidity of the product may be positively perceived by consumers as an expression of traditional food production processes; this has been confirmed using the high overall quality scores obtained for probiotic sausages. The decrease in the pH inhibits the growth and multiplication of undesirable microorganisms, including putrefactive bacteria, thus extending the shelf life [4].

Some studies have found 26 terpene compounds in dry fermented sausages, mainly due to the addition of spices [19,21]. The metabolism of microorganisms, including moulds and yeast, in the production of terpenes should be considered [23]. The total content of terpenes increased during storage in all the sausage samples studied.

Iacumin et al. [24] also observed an increase in terpene contents during the fermentation of Pitina sausages. According to the authors, the reason for this was the loss of water during the fermentation process, which increased the proportion of fat, in which terpenes are easily soluble. In the case of the control sausages after fermentation and after 12 weeks of storage, the level of terpenes, especially those of 3-carene and alpha-limonene, was higher than that in the sausages with probiotics. Different terpene concentrations, especially in sample groups P0 and K12 (Table 3), could be attributed at least partially to the sausage matrix effect. Thus, the terpenes were differentially released into the headspace at the end of storage and specifically in the control samples; this could be attributed to the different pH values (among other things). A partially similar effect was observed in the study by Carballo et al. [25].

Terpenes, such as alpha-limonene (citrus peel), 3-carene (raisin, sweet, terpene), alpha-terpinolene (sweet and pine), linalool (floral, lavender, citrus), terpinen-4-ol (pine), alpha-terpineol (floral, lilac), beta-elemene (herbal, spicy), beta-caryophyllene (clove, turpentine), and alpha-caryophyllene (orange, pepper), may have contributed to the aroma of the fermented sausages. In the case of the control sausages, the contents of alpha-terpinolene, linalool, terpinen-4-ol, and alpha-terpineol were higher than those in the sausages with probiotics. Owing to the large group of terpenes found in the study sausages, it is difficult to unequivocally assess their influence on the quality and sensory characteristics. In addition, the presence of terpenes results from the additives and spices used, affecting the perception of positive sensory features, such as fruity, herbal, pine, and spicy odour [26,27].

In the sausages with probiotics, the total level of aldehydes increased during the 12 weeks of storage and was significantly higher than that in the control sausages. The highest content at week 12 was found for the hexanal, octanal, and nonanal formed as a result of the oxidation of unsaturated fatty acids. Lipid oxidation processes take place in dry fermented meat products due to the participation of bacteria, including *Lactobacillus* spp., which leads to the formation of aldehydes, which, in turn, positively affect the sensory characteristics of the product [16,28]. The major contributors to the typical aroma of meat products during the ripening or dry-curing stages are the compounds of lipid oxidation [29]. Di Cagno et al. [27] found a similar high content of hexanal in traditional Italian sausages. Qi et al. [30] observed a significant increase in the hexanal content in Cantonese sausage (raw maturing sausages) during 30 days of storage. Octanal is characterised by meat, citrus, herbal, and floral odours. Nonanal is characterised by the rancid, soap, fat, plastic, and hexanal odours of green leaves [17,27,31,32]. Moreover, hexanal is considered an indicator of the proper course of the oxidation processes in the meat fermentation process [18].

The content of benzaldehyde in the samples with probiotics was higher after fermentation and during the entire storage period. The presence of benzoaldehyde in fermented sausages may be derived from phenylalanine by bacterial activity [16]. The odour of benzaldehyde is considered characteristic of fermented meat products and is described as cherry–marzipan, burnt sugar, and almond [4,33]. In a study of probiotic sausages during the 6th week of storage, a statistically higher score of dried meat odour was recorded, and this could have been due to the higher aldehyde content. Aldehydes are among the compounds that have a significant impact on the odour of the product as they have a low perception threshold [30,34].

In a study of probiotic sausages, the levels of esters, mainly those of ethyl acetate (fruity aroma) and ethyl butanoate (pineapple aroma), were significantly higher after fermentation and in the 6th week of storage. In contrast, the ethyl lactate and ethyl octanoate (fruity, apple, and floral aromas) levels were higher at each stage of the study in probiotic sausages. The higher ester concentrations in fermented sausages can be attributed to LAB esterase activity [35,36]. Esters are the aromatic compounds responsible for the fruity odour of dry fermented sausages [37]. Esters, due to their low sensory threshold, are believed to have a significant influence on the odour of meat products. In dry fermented sausages with and without probiotic starter cultures, the effect of esters during storage could mask the perception of negative sensory factors, which resulted in low scores for bitter, storage, and other flavours, as well as spicy and other odours. In addition, the fruity odour may mask the rancid odour of meat fat [24,37,38]. The increase in the ester content also contributed to the proper sausages aroma both with probiotics and the control [23]. In a study by Wen et al. [39], esters, such as ethyl acetate, ethyl lactate, methyl hexanoate, and ethyl phenylacetate, were found in traditional Chinese fermented sausages called Harbin, and this was associated with the increased activity of bacterial esterases. Ethyl butanoate has also been found in other meat products, such as smoked sausages and dry-cured loins [40].

The total content of ketones, especially acetoin, in the control sausages was higher after fermentation and 6 weeks of storage. During the 12th week of storage, acetoin was not found in the probiotic and control sausages. This may have been due to the presence of LAB, which were responsible for further metabolic changes, including the oxidation of dodiacetyl (2, 3-butanedione), which has a low perception threshold and a sweet buttery odour [41]. Ketones are formed during the metabolic changes of pyruvate. Methyl ketones have a low odour threshold and contribute to animal fat and fruity, fatty odours; however, owing to their high threshold of sensation, they do not have a significant effect on the aroma of the product [17,22,41].

The total alcohol content in the probiotic sausages was significantly higher in the 6th week of storage. On the other hand, the level of ethanol in the sausages with the probiotic starter culture was higher both after fermentation and during 6 weeks of storage. In meat fermentation, alcohols are produced through various metabolic carbohydrate pathways. One such pathway is the metabolic transformation of lactate with the participation of LAB [42,43]. The most abounded alcohol in the probiotic and control sausages was ethanol. The influence of alcohol on the sensory features is minor, probably due to the high threshold of sensation. [22].

The level of phenols (mainly phenol) was significantly higher in the control sausages after fermentation and during the 12-week storage period. Phenols are compounds that inhibit oxidative lipid breakdown and the growth of microorganisms [2]. The spices added in the sausage production process may be the source of the phenols [22]. Owing to their antioxidant and antimicrobial properties, phenols are considered promising substances to protect against meat spoilage. Natural phenols have beneficial effects on human health, in contrast with synthetic phenolic antioxidant substances, which have been linked to toxicity and carcinogenesis [44]. The phenols identified in sausages have at least two origins: the catabolism of aminoacids and added spices. The total content of the sulfur compounds decreased during storage compared with that after the fermentation in both variants of sausages. Similar results were reported by Carballo et al. [25]. Sulfur compounds can have a negative effect on the sensory characteristics of meat products and are responsible for the odours of meat and rancid meats [45]. The most abundant sulfur compounds present in the probiotic and control sausages were allyl methyl sulfide and diallyl disulfide, which are the garlic derived compounds [46].

### 2.3. Sensory Evaluation

**Figure 1:**
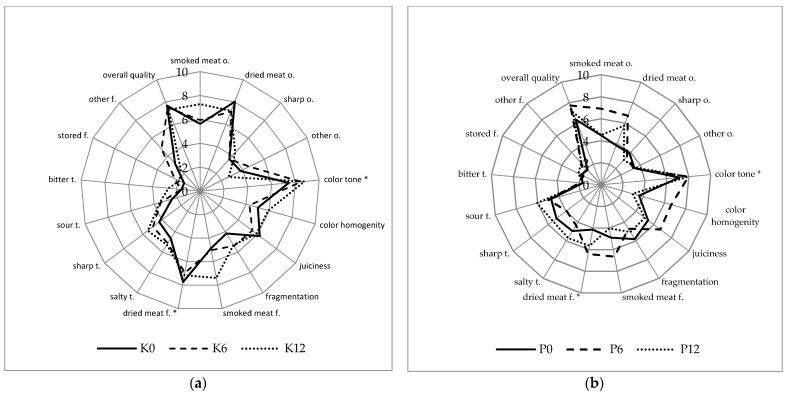
(**a**) Sensory quality characteristics of control dry fermented sausages without *L. rhamnosus* LOCK900 addition: after ripening process (K0), after 6 (K6), after 12 (K12) weeks of storage; (**b**) Sensory quality characteristics of probiotic dry fermented sausages with *L. rhamnosus* LOCK900 addition: after ripening process (P0), after 6 (P6) and after 12 (P12) weeks of storage. c—colour, o—odour, f—flavour, t—taste; * Values are significantly different (α ≤ 0.05).

The processes of the fermentation and maturation of meat products are primarily aimed at insuring product safety, improving the flavour, odour, texture, and colour, and extending the storage time. It is expected that the addition of probiotic microorganisms will also have a positive effect on the aforementioned features and provide a health-promoting effect, increasing the attractiveness of the product offered to consumers. The general sensory quality of the probiotic and control sausages was high both after the maturation and during storage. The overall quality of the probiotic sausages was rated the highest, with a score of 7.7 in the 6th week of storage; in the 12th week, it slightly decreased to 7.1 (Figure 1b). After the fermentation and during storage, no statistically significant differences were found in the overall quality assessment between the sausages with LAB and control sausages.

The high overall quality of the control and probiotic sausages after fermentation and during storage can be attributed to the high scores of positive sensory factors, such as smoky odour, dried meat odour, colour tone, and dried meat flavour (Figure 1a,b).

**Figure 2:**
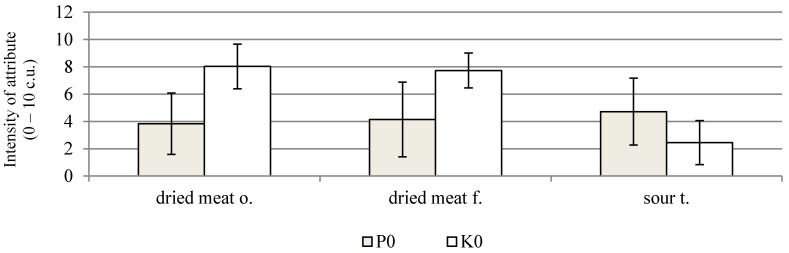
Significant differences in sensory attributes between dry fermented sausages with (P0) and without (K0) *L. rhamnosus* LOCK900 addition after fermentation process; (α ≤ 0.05); abbreviations as at Figure 1.

**Figure 3:**
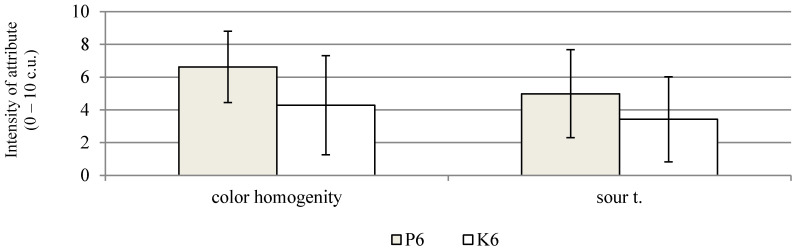
Significant differences in sensory attributes between dry fermented sausages with (P6) and without (K6) *L. rhamnosus* LOCK900 addition after 6 weeks of storage; (α ≤ 0.05); abbreviations as at Figure 1.

**Figure 4:**
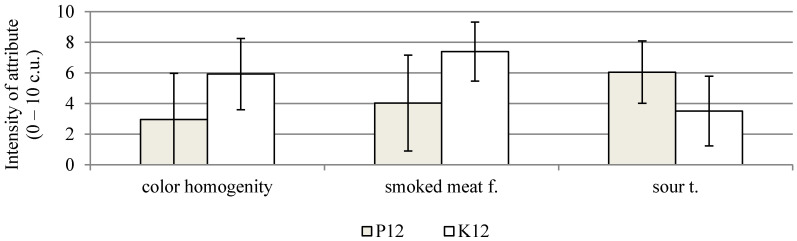
Significant differences in sensory attributes between dry fermented sausages with (P12) and without (K12) *L. rhamnosus* LOCK900 addition after 12 weeks of storage; (α ≤ 0.05); abbreviations as at Figure 1.

On the other hand, negative sensory features, such as the perception of bitter flavour, storage and other flavours, and spicy and other odours, show a low intensity. In the 6th week of the storage of the probiotic sausages, a statistically significant increase in the ratings for dried meat odour, colour tone, colour homogeneity, dried meat flavour, and smoked flavour was found (Figure 1b). In the 12th week of storage, the range of changes was smaller, and a statistically significant decrease was recorded only for the dried meat flavour. Similar positive sensory assessments were performed by Radulović et al. [47]. After 40 days of storage, dry fermented sausages with *Lactobacillus helveticus* RO52 and *Bifidobacterium longum* RO175, as well as *Pediococcus pentosaceus* and *Staphylococcus xylosus* strains, in most variants, received sensory scores above 7 on a 9-point scale. A comparison of the sensory qualities of both the study products, made after the fermentation process, showed that the control sausages were characterised by a significantly higher score for dried meat odour and flavour, and a lower score for sour flavour (Figure 2). In the 6th week of storage, a comparison of both the products showed a significant increase in the assessment of colour homogenity and higher values for the sour flavour of sausages with probiotic strains (Figure 3). In the case of the sausages without probiotics, a statistically significant increase in colour tone was noted in the 6th and 12th weeks of storage, and, in the 12th week, the evaluation of the dried meat flavour was significantly more positive (Figure 1a). After 12 weeks of storage, the probiotic sausages showed significantly lower scores in terms of colour homogenity and smoked flavour compared with the product from the control group (Figure 4). The higher score for sour flavour in the 12th week of storage can be explained by the growing number of LAB cells in the probiotic sausages. This did not affect the general quality scores, which were similar for both study products (Figure 1a,b). These results are concordant with those of Chen et al. [48]. They concluded that the use of a multi-strain starter, including *S. xylosus* SX16 and *L. plantarum* CMRC6, accelerated the acidification and proteolysis during ripening, improving the microbiological safety and sensory attributes of the fermented dry sausage. According to other studies, LAB bacteria may adversely affect the flavour profile. In the studies by Bedia et al. [49], it was found that the addition of *Lactobacillus sakei* and *Pediococcus pentosaceus* to fermented pork salami was responsible for excessive acidity, and it was determined that the pH of the product should not be lower than 5.

## 3. Materials and Methods

### 3.1. Probiotic Starter Culture

The strain *L. rhamnosus* LOCK900 is known to fulfil the criteria required for probiotic bacteria. LOCK900 (patent no.: P.382760; strain deposit number: CP005454) was obtained from the collection of the Technical University of Łódź in Poland [50]. The probiotic starter culture was prepared according to the methods of Neffe-Skocińska et al. [51]. The exact procedure for preparing a probiotic starter culture for the production of dry fermented meat products is the subject of Polish patent number 226236. The number of LOCK900 cells in the prepared liquid starter culture was 9 log CFU mL^−1^. Bacteria were added to the raw meat at a concentration of 2 mL kg^−^^1^ of meat input. The dry fermented sausages used in this study were from the same test batch; hence, it was certain that LOCK900 was present and dominated the natural lactic microflora of the tested meat products. The presence and survival of the LOCK900 strain in the tested sausages after 21 days of ripening process and after 12 weeks of refrigerated storage in approximately 90% was confirmed by phenotypic and genetic methods according to the studies of Neffe-Skocińska et al. [6].

### 3.2. Dry Fermented Sausage Manufacturing

Sausages were manufactured in a local Polish meat processing plant (Agro-Visbek Inc., Nakło, Poland) with the technology previously used in laboratory conditions [51,52,53].

The sausages were prepared from musculus biceps femoris and pork backfat at a ratio of 70:30 (*w*/*w*). The meat was purchased from a meat processor at 48 h post-mortem (cross-breeds of the Puławska Polish Landrace, Nakło, Poland) with a body weight of approximately 120–130 kg at slaughter. The meat was cured using a curing mixture (99.5% sea salt, 0.5% sodium nitrite) in amount of 28 g/kg in relation to the meat. The pork meat was then stored at 2 °C for 24 h. Two batches of 300 kg ingredients for the sausages were prepared; they comprised the control samples of dry fermented sausages without addition (control sausages) and a product treated with probiotic starter culture of *L. rhamnosus* LOCK900 (probiotic sausages) (Table 4).

One portion of cured pork meat was mixed with 50 g kg^−^^1^ of cold water, glucose (5 g kg^−^^1^ of meat), and spices: garlic powder (5 g kg^−^^1^ of meat), black pepper (5 g kg^−^^1^ of meat), and marjoram (3 g kg^−^^1^ of meat). The second portion was mixed separately with the probiotic starter culture (2 mL kg^−^^1^ of meat, containing 9 log CFU mL^−^^1^). The probiotic starter culture was prepared in the laboratory and transported for meat processing in 4 h. The ingredients were added and mixed, and then stuffed into natural casings. The sausages were matured under the following conditions: 24 h at 18 °C and 85–92% relative humidity, followed by a gradual decrease in temperature to 17–16 °C and relative humidity of 75–80%. The products were then maintained under these conditions for 19 days, for a total of 21 days of fermentation. The fermentation time was 21 days. After 4 days of fermentation, the sausages were cold-smoked (30 min at 30 °C). After 21 days of fermentation, the sausages were vacuum-packed and stored in a refrigerator (4 °C) for 84 days. Microbiological, physical, and chemical analyses were performed after 21 days of fermentation and after 6 and 12 weeks of storage. The entire experiment was repeated thrice.

### 3.3. pH Determination

The pH value of filtrates was measured by mixing 10 g of a minced sample with 50 mL of deionised water for 1 min using a homogeniser (Bamix 200, Greifensee, Switzerland). The pH was measured using a digital pH meter (Seven Compact S220, Mettler Toledo, Greifensee, Switzerland) equipped with a pH electrode (Seven Compact S220, InLab Cool, Mettler Toledo, Greifensee, Switzerland). The pH readings were recorded exactly 4 min after the insertion of the electrode into the sample. The pH meter was standardised with buffer solutions at pH 2.0, 4.0, 7.0, and 10.0.

### 3.4. Microbiological Analyses

Microbiological analyses of sausages were carried out immediately after the meat ripening period (time 0) of 6 and 12 weeks of refrigerated storage under anaerobic conditions. The study was carried out with a traditional plate method using MRS agar (Merck, Germany), which was similar to dedicated culture media for lactic acid bacteria (LAB). The incubation parameters were 37 °C for 48 h. All the microbiological determinations were carried out in accordance with the global ISO guidelines.

### 3.5. Volatile Compound Analyses

The analysis of volatile organic compounds from sample headspaces was performed using solid-phase microextraction (SPME) and gas chromatography coupled with mass spectrometry (GC–MS). Volatile compounds were extracted from headspaces using DVB/CAR/PDMS SPME fibre (divinylbenzene/carboxen/polydimethylsiloxane, 1 cm length, 50/30 μm thickness; Supelco, Bellefonte, PA, USA). Before analysis, the fibre was conditioned by heating in a GC injection port at 270 °C, and approximately 5 g of the homogenised sample was placed in a 20 mL vial, closed with a silicone–Teflon sealing cap, and heated to 37 °C for 1 h in order to stabilise the concentrations of volatile compounds in the vial. Then, the SPME fibre was introduced into the sample headspace for a period of 40 min. Thereafter, the fibre was quickly transferred to the GC injection port (splitless mode, temperature of 270 °C) to desorb volatiles into the GC system. Chromatographic separation was performed using an Agilent 6890 GC system (Agilent Technologies, Palo Alto, CA, USA) coupled with an Agilent 5795 quadruple MS instrument (Agilent Technologies). A DB-5MS column (30 m, 0.25 mm, 0.25 µm, 5% diphenyl-95%-polydimethylsiloxane, Agilent Technologies, Palo Alto, CA, USA) was used with helium as the carrier gas at a flow rate of 0.9 mL/min. The GC oven was programmed as follows: an initial temperature of 38 °C was maintained for 10 min, increased to 200 °C at a rate of 4 °C/min, maintained for 2 min, and then raised to 250 °C at a rate of 20 °C/min, and the final temperature (250 °C) was maintained for 7 min. The mass spectrometer was programmed as follows: the temperatures of the ion source and mass analyser were 230 and 150 °C, respectively, and mass spectra were recorded in electron ionisation (EI) mode with an ionisation energy of 70 eV and an ion scanning range of 33–350 *m*/*z* (amu). The obtained spectra were subsequently analysed using the Mass Selective Detector ChemStation program (D.02.00.237, Agilent Technologies, Palo Alto, CA, USA). Individual peak identification was based on the comparison of the mass spectra with references in the mass spectra databases of NIST 08 (US National Institute of Standards and Technology) and Wiley 8th Ed. Compound mass spectra targets were confirmed by comparing linear retention indices (LRIs) calculated relative to C6–C20 alkanes with an LRI database built in the NIST.08 mass spectra library. In order to use LRI method, a C8–C20 alkanes mixture (Fluka, Buchs, Switzerland) and pure standards of hexane and heptane (Sigma Aldrich, St. Louis, MI, USA) were analysed. Quantity of each compound was determined as a peak percentage area relative to total area of all peaks on the chromatogram (relative content (%)) [54].

### 3.6. Sensory Analyses

The sensory quantitative descriptive analysis method (ISO 13299:2016-05) [55] was used with an unstructured, linear graphical scale: 100 mm was converted to numerical values (0–10 conventional units, where 0 means absence or very low intensity of the descriptor and 10 means very high intensity of the descriptor). Descriptors were chosen and defined during a panel discussion and then verified in a preliminary session. Finally, a list of 17 sensory attributes with definitions was used by the panel: four for odour, two for colour, one for appearance, one for texture by mouth, and eight for flavour and overall sensory quality. Sensory analyses were performed by a panel of 8 trained persons (5 women and 3 men, aged 30–45 years) according to the ISO 8586:2014-03 procedure [56]. The panelists were selected for their sensory ability and experience with the sensory evaluation process of meat products. They possessed the necessary skills to describe the flavour, taste, and odour attributes of different samples. They underwent descriptive tests with the use of a series of food products that included meat fermented products in which they described the sensory characteristics of the samples. During preliminary evaluation sessions, the proper understanding of the given set of attributes by the panelists was confirmed. The dry fermented sausages were sliced to an approximately 2 mm thickness with an electric slicing machine and placed in plastic odourless transparent boxes covered with lids. All the samples for evaluation were individually coded with three-digit codes and delivered at random sequence to limit the impact of the carryover effect (i.e., the impact of a previous sample on a subsequent sample). The evaluation was repeated twice so that each mean result was based on a minimum of 16 unitary results while carrying out sensory assessments, constant temperature, lighting, and elimination of distracting factors, such as noise and off odours, were applied. A trained panel was used to evaluate the sensory quality of sausages after fermentation and 6 and 12 weeks of storage under refrigeration (4 °C).

### 3.7. Statistical Analyses

Three independent series of experiments (replicates) were performed under industrial conditions (for every production process, a random selection of raw meat material was used) at different time points, and a completely randomised design was used. All the analyses were performed at least in triplicate (*n* = 6). A one-way analysis of variance (ANOVA) was performed to determine occurrence of significant differences between means of compared groups K0, K6, K12, P0, P6, P12. Next, post-hoc Tukey’s test was used to determine specifically which groups differed. Differences were considered significant at α ≤ 0.05. All the data were analysed using Statistica version 13.1 (Statsoft Inc., Cracow, Poland).

## 4. Conclusions

Dry fermented pork sausages with *L. rhamnosus* LOCK900 starter probiotic culture were characterised by positive sensory features, such as smoky and dried meat odour, better colour tone, juiciness, and dried meat flavour, and the overall quality remained at a high level during storage and did not differ from that of the control sausages.

The addition of *L. rhamnosus* LOCK900 probiotic starter culture increased the levels of acidic volatile compounds, aldehydes, and esters in the dry fermented pork sausages. During the storage of the probiotic sausages, an increase in the scores was noted for the discriminants positively associated with products of natural origin, such as dried meat smell, colour tone, colour homogenity, and the dried meat and smoked flavours. Dry fermented pork sausages with *L. rhamnosus* LOCK900 probiotic starter culture are attractive products for consumers that combine the potential health-promoting effects of probiotics with the flavour of traditional food.

## Figures and Tables

**Table 1:** The count of LAB of dry fermented pork sausages after fermentation after 6 and 12 weeks of storage (log CFU g^−1^).

Kind of Sample	After Fermentation	Week 6	Week 12
CONTROL SAUSAGES	6.89 aA ± 0.31	7.45 aA ± 0.96	7.11 bA ± 0.62
PROBIOTIC SAUSAGES	8.12 aB ± 0.67	8.43 bB ± 0.88	8.67 aB ± 0.32

Explanatory notes: Number of LAB: Data are expressed as mean ± SD. Means in the same column followed by different uppercase letters represent significant differences (α ≤ 0.05). Means in the same row followed by different lowercase letters represent significant differences (α ≤ 0.05).

**Table 2:** pH values of dry fermented pork sausages after fermentation after 6 and 12 weeks of storage.

Kind of Sample	After Fermentation	Week 6	Week 12
CONTROL SAUSAGES	5.44 bB ± 0.05	5.31 aB ± 0.02	5.28 aB ± 0.02
PROBIOTIC SAUSAGES	4.95 bA ± 0.03	4.88 aA ± 0.10	4.70 aA ± 0.02

Explanatory notes: Data are expressed as mean ± SD. Means in the same column followed by different uppercase letters represent significant differences (α ≤ 0.05). Means in the same row followed by different lowercase letters represent significant differences (α ≤ 0.05).

**Table 3:** Relative content (%) of volatile organic compounds in CONTROL and PROBIOTIC sausages after ripening after 6 and 12 weeks of storage.

Compound Name	K0	K6	K12	P0	P6	P12
Acetic acid	2.90 a	4.15 b	1.86 d	5.20 c	4.85 bc	3.02 a
Butyric acid (Butanoic acid)	0.00 a	0.00 a	0.00 a	0.11 b	0.15 b	0.03 a
Total acids	2.90 a	4.15 d	1.86 c	5.31 b	5.00 b	3.05 a
Ethanol	1.43 a	1.57 ab	2.04 bc	2.19 c	2.92 d	1.63 abc
2-ethylcyclobutan-1-ol	0.05 a	0.02 a	0.02 a	0.17 b	0.00 a	0.00 a
2-Furanmethanol (Furfurylalcohol)	0.70 b	0.72 b	0.39 c	0.20a	0.09 a	0.19 a
1-Octanol	0.00 a	0.00 a	0.00 a	0.00a	0.00 a	0.05 b
Total alcohols	2.17 ab	2.31 ab	2.45 abc	2.56 bc	3.01 c	1.87 a
Hexanal	0.23 a	0.17 a	0.15 a	0.27a	0.24 a	0.72 b
Heptanal	0.09 a	0.04 a	0.03 a	0.08 a	0.05 a	0.61 b
Benzaldehyde	0.05 a	0.07 a	0.11 b	0.15c	0.11 b	0.18 c
Octanal	0.27 b	0.02 a	0.00 a	0.22 ab	0.17 ab	1.02 c
Benzeneacetaldehyde	0.09 ab	0.11 a	0.08 ab	0.07 ab	0.06 b	0.12 a
Nonanal	0.55 a	0.31 a	0.21 a	0.39 a	0.43 a	2.23 b
Total aldehydes	1.26 a	0.72 a	0.57 a	1.19 a	1.07 a	4.87 b
Dimethylamine	0.00 a	0.00 a	0.04 a	0.24 b	0.15 ab	0.07 ab
Total amines	0.00 a	0.00 a	0.04 a	0.24 b	0.15 ab	0.07 ab
Toluene	0.07	0.10	0.10	0.08	0.07	0.05
Total aromatic hydrocarbons	0.07	0.10	0.10	0.08	0.07	0.05
Acetic acid ethyl ester (Ethyl Acetate)	1.55 a	2.23 a	1.85 a	3.10 b	3.19 b	2.01 a
Propanoic acid, 2-methyl-, ethyl ester (Ethyl isobutyrate)	0.06	0.08	0.07	0.05	0.03	0.02
Butanoic acid, ethyl ester (Ethyl butanoate)	0.24 a	0.31 ab	0.41 b	0.80 d	0.73 cd	0.69 c
Lactic acid, ethyl ester (Ethyl lactate)	0.00 a	0.01 a	0.10 c	0.33 b	0.37 b	0.22 d
Butanoic acid, 2-methyl-, ethyl ester (Ethyl 2-methylbutyrate)	0.05	0.07	0.06	0.06	0.03	0.03
Octanoic acid, ethyl ester (Ethyl octanoate)	0.03 c	0.08 b	0.16 a	0.09 b	0.14 a	0.13 a
Linalylacetate (Bergamiol)	0.07 a	0.09 ab	0.09 ab	0.08 a	0.13 b	0.14 b
Decanoic acid, ethyl ester (Ethyl decanoate)	0.00 c	0.03 a	0.10 d	0.02 a	0.08 b	0.07 b
Total esters	1.99 a	2.89 ab	2.83 ab	4.52 c	4.69 c	3.31 b
2,3-butanediol	0.09 ab	0.17 b	0.00 a	0.00 a	0.00 a	0.00 a
Total glycols	0.09 ab	0.17 b	0.00 a	0.00 a	0.00 a	0.00 a
Acetoin (3-hydroxy-2-butanone)	0.82 c	0.53 b	0.00 a	0.13 a	0.00 a	0.00 a
2-Methyl-2-cyclopenten-1-one	0.16 a	0.13 a	0.14 a	0.09 ab	0.05 b	0.11 ab
3-Methyl-2-cyclopenten-1-one	0.14 b	0.15 b	0.09 c	0.05 a	0.05 a	0.05 a
2,3-Dimethyl-2-cyclopenten-1-one	0.16 b	0.17 b	0.11 c	0.03 a	0.00 a	0.00 a
2-Nonanone	0.10 c	0.10 c	0.06 b	0.03 ab	0.00 a	0.00 a
Total ketones	1.38 d	1.07 c	0.40 b	0.32 ab	0.10 a	0.16 ab
Phenol	0.19 b	0.22 b	0.15 c	0.11 a	0.12 a	0.10 a
Creosol (2-Methoxy-4-methylphenol)	0.21	0.28	0.21	0.05	0.07	0.06
m-Cresol (Phenol, 3-methyl)	0.03 ab	0.23 c	0.11 b	0.00 a	0.00 a	0.00 a
Total phenols	0.43 b	0.73 c	0.47 b	0.16 a	0.18 a	0.16 a
Allylthiol	1.51 ab	0.00 d	1.09 a	3.79 e	2.51 c	2.31 bc
Allyl methyl sulfide	3.08 b	1.95 a	1.93 a	2.63 ab	1.88 a	1.75 a
Dimethyl disulfide	0.08	0.07	0.05	0.24	0.07	0.10
Diallyl sulfide (Allylsulfide)	0.63 ab	0.61 ab	0.77 b	0.65 ab	0.50 ac	0.44 c
Allyl methyl disulfide	0.73 c	0.49 b	0.28 a	0.46 ab	0.30 a	0.38 ab
Diallyl disulfide	2.23 c	1.78 b	1.53 ab	1.37 a	1.13 a	1.26 a
Total sulfur compounds	8.26 bc	4.89 a	5.64 a	9.13c	6.39 ab	6.24 ab
Alpha-Thujene (3-Thujene)	2.23 b	2.15 b	2.67 ab	2.91 a	2.88 a	2.81 a
Alpha-Pinene (2-pinene)	2.30 ab	2.14 a	2.28 a	3.32 d	2.67 bc	2.78 c
Camphene	0.14 ab	0.13 ab	0.13 ab	0.18c	0.12 a	0.14 b
Sabinene (4(10)Thujene)	6.08 ab	5.55 a	6.02 ab	7.46 b	6.94 ab	6.56 ab
Beta-Pinene	2.63 a	2.33 a	2.51 a	3.44 b	2.85 ab	2.71 a
Beta-Myrcene	3.06 ab	3.54 a	4.09 c	2.57 b	3.19 a	3.28 a
Alpha-Phellandrene	3.52 ab	3.87 b	4.44 d	2.49 c	3.18 a	3.37 a
3-Carene	14.58 a	14.82 a	13.62 ac	11.18 b	12.05 bc	12.78 abc
Alpha-Terpinene	1.82 a	1.97 ab	2.16 bc	1.90 a	2.35 c	2.20 bc
o-Cymene	0.09	0.10	0.08	0.10	0.09	0.11
p-Cymene	2.54 ab	2.54 ab	2.39 b	2.82 ac	2.79 ac	2.92 c
Alpha-Limonene	19.33 a	20.14 ab	22.60 b	17.81 a	19.25 a	18.81 a
Beta-Ocimene	0.13 a	0.12 a	0.19 b	0.09 a	0.10 a	0.12 a
Gamma-Terpinene	2.44 a	2.67 ab	3.12 c	2.65 ab	3.26 c	2.95 bc
4-thujanol (Sabinenehydrate)	0.57 a	0.58 a	0.48 ab	0.39 b	0.48 ab	0.50 ab
Alpha-Terpinolen	1.77 c	1.91 cd	1.94 d	1.33 a	1.49 ab	1.59 b
Linalol	0.83 a	0.82 a	0.74 a	0.46 c	0.57 b	0.60 b
Terpinen-4-ol	1.22 a	1.20 a	1.21 a	0.93 b	1.26 a	1.13 ab
Alpha-Terpineol	0.29 d	0.27 c	0.23 bc	0.17 a	0.20 ab	0.22 abc
Alpha-Copaene	0.16 ab	0.14 a	0.17 ab	0.16 ab	0.19 bc	0.21 c
Beta-Elemene	0.12 a	0.10 a	0.11 a	0.07 b	0.10 a	0.11 a
Beta-Caryophyllene	5.38 a	4.66 a	5.24 a	3.42 b	4.95 a	4.93 a
Alpha-Caryophyllene	0.24 a	0.19 ab	0.22 a	0.15 b	0.21 a	0.22 a
Beta-Selinene	0.12 a	0.11 ab	0.11ab	0.09 b	0.11 ab	0.13 a
Alpha-Selinene	0.09 a	0.08 a	0.09 a	0.06 b	0.08 a	0.09 a
Total terpenes	71.64 a	72.12 a	76.83 c	66.12 b	71.33 a	71.27 a

Explanatory notes: Control sausages without *L. rhamnosus* LOCK900 addition: K0-after ripening process, K6-after 6 weeks of storage, K12-after 12 weeks of storage. Probiotic sausages with *L. rhamnosus* LOCK900 addition: P0-after ripening process, P6-after 6 weeks of storage, P12-after 12 weeks of storage. Means in the same row followed by different letters represent significant differences (α ≤ 0.05).

**Table 4:** Kinds and markings of dry fermented pork sausages used in the experiment.

Kind of Sample	After Fermentation	After 6 Weeks of Storage	After 12 Weeks of Storage
CONTROL SAUSAGES	K0	K6	K12
PROBIOTIC SAUSAGES	P0	P6	P12

## Data Availability

Not applicable.
